# Role of HER2 in Response to Neoadjuvant Endocrine Therapy in Luminal Breast Cancer

**DOI:** 10.3390/curroncol33020099

**Published:** 2026-02-04

**Authors:** Celia del Monte, Covadonga Martí, Elena Rodríguez, Elisa Moreno-Palacios, Laura Frías, Marcos Meléndez, Adolfo Loayza, Laura Yébenes, José Ignacio Sánchez-Méndez

**Affiliations:** 1 Department of Obstetrics and Gynecology, Faculty of Medicine, Universidad Autónoma de Madrid, 28029 Madrid, Spain; celia.delmonte@salud.madrid.org (C.d.M.); joseignacio.sanchez@salud.madrid.org (J.I.S.-M.); 2 Breast Unit, Obstetrics and Gynecology Department, University Hospital La Paz, 28046 Madrid, Spain; elena.rodriguezg@scsalud.es (E.R.); elisa.moreno@salud.madrid.org (E.M.-P.); lfrias@salud.madrid.org (L.F.); marcos.melendez@salud.madrid.org (M.M.); adolfo.loayza@salud.madrid.org (A.L.); 3 Breast Unit, Pathology Department, University Hospital La Paz, 28046 Madrid, Spain; laura.yebenes@salud.madrid.org; 4 Hospital La Paz Institute for Health Research (IdiPAZ), 28029 Madrid, Spain

**Keywords:** breast cancer, luminal, HER2-low, endocrine therapy, neoadjuvant

## Abstract

**Purpose**: Recently, authors have shown increasing interest in the emerging HER2-low subtype in breast cancer. These tumors are currently classified and treated as HER2-negative tumors. Neoadjuvant endocrine therapy (NET) has been used to achieve tumor downstaging and assess treatment response in postmenopausal women with low-grade luminal HER2-negative tumors. This study aimed to determine whether the response to NET was affected by low levels of HER2 expression in luminal tumors. **Methods**: The study was a single-centered retrospective investigation. Data were gathered from the medical records of patients from 2017 to 2023. All patients had luminal HER2-negative tumors and were treated with NET and subsequent surgery. **Results**: In total, 175 tumors were analyzed; 24.0% of the tumors were HER2-zero, 48.0% were HER2-low 1+, and 28.0% were HER2-low 2+. No significant differences were found when assessing NET response between the three groups, nor when evaluating tumor features. Response to NET was influenced by estrogen receptor levels, histological subtype and histological grade at diagnosis, Ki67 levels at interim biopsy, and NET duration. No differences were found for progression-free survival (PFS) or overall survival (OS) between HER2 groups. The group with Ki67 ≤ 10% at interim biopsy had longer PFS than the Ki67 > 10% group (*p* < 0.05). No differences for OS were found between these groups. **Conclusions**: Response to NET was not influenced by low levels of HER2 expression in luminal tumors when compared to HER2-zero tumors. PFS was longer for patients who had lower Ki67 levels at interim biopsy after NET.

## Simple Summary

Interest in the new HER2-low subtype of breast cancer is increasing. Currently, this subtype is classified and treated the same as HER2-negative tumors. This study aimed to determine whether response to neoadjuvant endocrine therapy (NET) is affected by low levels of HER2 expression in luminal tumors. Our findings suggest that low HER2 expression does not significantly impact the response to neoadjuvant endocrine therapy (NET) in luminal tumors compared to HER2-zero cases. Additionally, it does not affect other clinical or histopathological features. Rather, NET response may be influenced by factors such as estrogen receptor (ER) levels, histological subtype and grade, and early Ki67 changes. A decrease in Ki67 after short-term NET may be associated with longer progression-free survival (PFS).

## 1. Introduction

Breast cancer (BC) is the most frequently diagnosed cancer and the leading cause of cancer-related mortality among women worldwide [1]. Tumors are classified by histological features and immunohistochemistry (IHC) to guide treatment and prognosis [2,3]. Morphologically, invasive ductal carcinoma (IDC) accounts for 70–75% of cases, invasive lobular carcinoma (ILC) accounts for 12–15%, and rare subtypes account for 0.5–5% [2]. Perou et al. [4] first linked tumor transcriptomes with molecular subtypes. Based on the IHC for estrogen receptor (ER), progesterone receptor (PR), human epidermal growth factor receptor 2 (HER2), and Ki67, four intrinsic molecular subtypes have been defined: Luminal A (ER-positive/HER2-negative with high PR and low Ki67), Luminal B (ER-positive with high Ki67 and/or low PR; HER2 may be overexpressed), HER2-positive (HER2-positive, ER-negative/PR-negative), and basal-like/triple-negative (ER-negative/PR-negative/HER2-negative, variable Ki67) [2,3].

**Figure 1:**
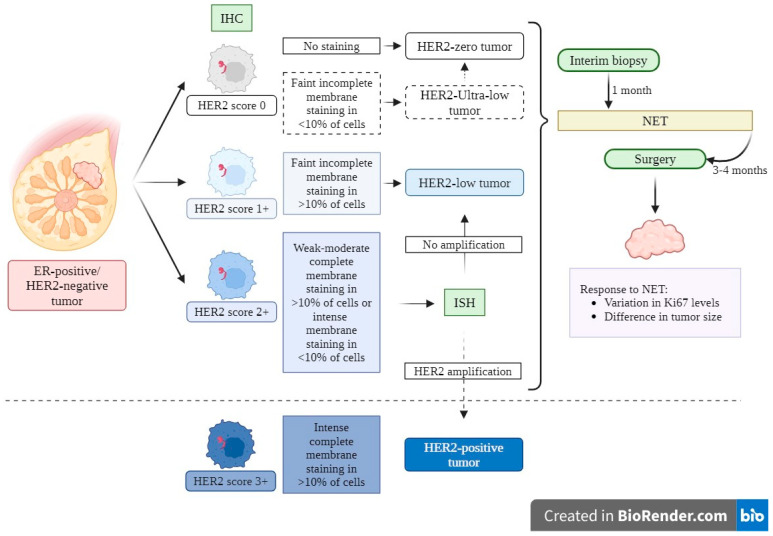
Current classification and treatment strategy for ER-positive/HER2-negative tumors. A score of 0 in IHC is categorized as HER2-zero, and a score of 1+ and 2+ with no amplification in ISH are classified as HER2-low. A score of 3 in IHC and 2+ with amplification in ISH categorises the tumor as HER2-positive. In this study, NET was given to all tumors. An interim biopsy was performed after 1 month of NET, and surgery was performed at least 3–4 months after diagnosis. NET response was measured by variation in Ki67 levels and difference in tumor size. Abbreviations: ER, estrogen receptor; HER2, human epidermal growth factor receptor 2; IHC, immunohistochemistry; ISH, in situ hybridization; NET, neoadjuvant endocrine therapy. Created with BioRender.com.

HER2-positive tumors overexpress the HER2 protein, a cell membrane tyrosine kinase that activates proliferative signaling pathways via receptor dimerization [5,6]. This confers a more aggressive behavior, whilst also enabling HER2-targeted therapy [6,7,8]. HER2-positivity is defined as IHC 3+ (intense complete membrane staining in >10% of cells) or gene amplification on in situ hybridization (ISH). Tumors with IHC scores of 0 (no staining or faint incomplete membrane staining in <10% of cells), 1+ (faint incomplete membrane staining in >10% of cells), or 2+ (weak-moderate complete membrane staining in >10% of cells or intense membrane staining in <10% of cells), and negative ISH are considered HER2-negative [6,7,9]. Recently, tumors with IHC-score 1+ or 2+ and no ISH amplification have been termed “HER2-low”, while IHC-score 0 tumors are “HER2-zero”; though both currently remain categorized as HER2-negative [7,10]. Some authors propose an additional “HER2-ultra-low” category for tumors with minimal staining in <10% of cells [6] (Figure 1).

Neoadjuvant treatment (NAT) has gained importance in BC, offering benefits such as tumor downsizing to allow for surgery and enabling breast-conserving procedures [2,3,11]. Based on tumor biology, NAT may include chemotherapy (CT), endocrine therapy (ET), or anti-HER2 agents. It also provides prognostic value by evaluating clinical and histopathological response [12]. Namely, proliferation marker Ki67 is used to guide NAT and assess prognosis. A Ki67 index ≤ 5% indicates low proliferation (CT not recommended), while ≥30% suggests high proliferation (CT advised), and intermediate values warrant further testing (e.g., gene-expression assays) [11,13].

Luminal tumors constitute around three-quarters of BC subtypes [8]. Among these, about 15% overexpress HER2 (ER-positive/HER2-positive) [14]. Accordingly, roughly two-thirds of all BC are ER-positive/HER2-negative tumors [12]. In recent years, neoadjuvant endocrine therapy (NET) has gained significant recognition and is now endorsed by current clinical practice guidelines. Nonetheless, indications for its use remain limited and are restricted to postmenopausal women with low-grade ER+/HER2-negative tumors [2,11]. Aromatase inhibitors are preferred, with treatment lasting at least 3–4 months [2,11,12,15]. NET response can be assessed by tumor size reduction and changes in Ki67 expression [12,16]. Notably, Ki67 levels after two weeks of NET better predicted outcomes than baseline values, combining prognostic and treatment-response information [13,17]. The Preoperative Endocrine Prognostic Index (PEPI) score uses tumor size, node status, ER status, and Ki67 levels in the surgical specimen to stratify recurrence risk [12,18,19,20].

Given their ER-positive/HER2-negative classification, luminal HER2-low tumors are currently treated with NET. However, new evidence supports the distinct biological behavior of HER2-low tumors, and trials are exploring and establishing novel therapies for this new emerging subtype. Treatment with anti-HER2 agents is already being administered in the metastatic setting for these tumors [21]. This study aimed to assess whether low HER2 expression (IHC-score 1+/2+ and negative ISH) impacts the response to NET in luminal BC, compared to HER2-zero tumors (IHC-score 0).

## 2. Materials and Methods

### 2.1. Study Design

**Figure 2:**
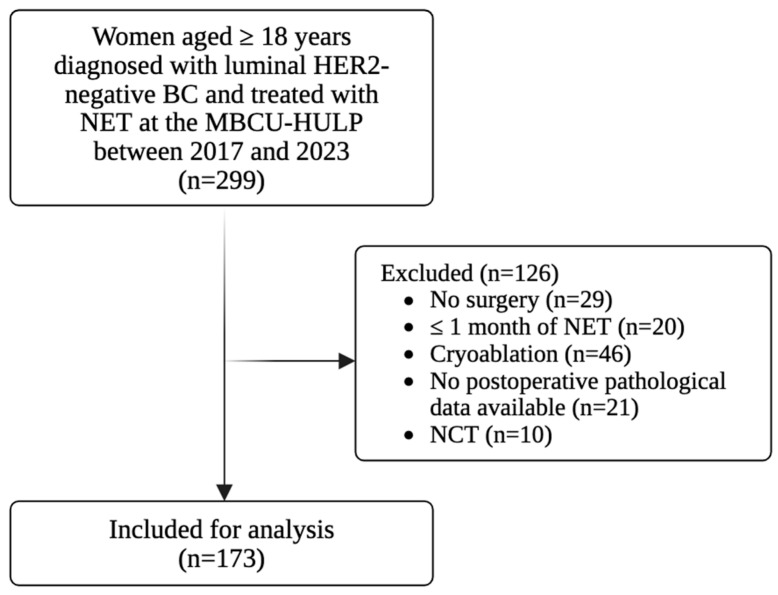
STROBE-flow diagram. Women aged 18 years or older diagnosed with luminal HER2-negative BC and treated with NET at the MBCU-HULP between 2017 and 2023 were screened. Patients who didn’t undergo surgery, received less than one month of NET, underwent cryoablation procedure, had no postoperative pathological data available or received NCT were excluded from the study. Abbreviations: BC, breast cancer; HER2, human epidermal growth factor receptor 2; MBCU, Multidisciplinary Breast Pathology Unit; NCT, neoadjuvant chemotherapy; NET, neoadjuvant endocrine therapy. Created with BioRender.com.

The study is a single-centered, retrospective, and observational investigation. Data were collected from the medical records of patients diagnosed with luminal HER2-negative breast tumors and treated with neoadjuvant hormonal therapy from 2017 to 2023. Inclusion criteria were as follows: (1) women aged 18 years and older; (2) diagnosed with luminal HER2-negative BC and treated with NET, as per evidence-based recommendations; (3) patients treated at the Multidisciplinary Breast Pathology Unit of the HULP between 2017 and 2023. Exclusion criteria included the following: (1) patients who did not undergo surgery, as postoperative pathological data were essential for the study endpoints, and neoadjuvant endocrine therapy inherently implies subsequent surgery; (2) patients who received less than one month of NET, typically due to institutional protocol (small tumor size warranting early surgery), patient preference for prompt operation, or recent enrollment before completing four weeks of therapy; (3) patients who underwent cryoablation procedure instead of surgery; (4) patients who had missing postoperative pathological data available (more than one measured variable); (5) patients who received neoadjuvant chemotherapy (NCT) (Figure 2).

### 2.2. Variables

The variables collected in this study included clinical, radiological, histopathological, therapeutic, and outcome-related data. Clinical features at diagnosis comprised the patient’s age (years). Radiological tumor characteristics included tumor size (mm) and clinical nodal status (cN; negative or positive). Histopathological features at diagnosis encompassed histological subtype (IDC, ILC, or other), ER levels (%), PR levels (%), Ki67 index (%), HER2 expression (0, 1+, or 2+), and histological grade (G1, G2, or G3). At interim, core needle biopsy (CNB), ER, PR, and Ki67 levels (%) as well as histological grade were reassessed. Therapeutic variables included duration of NET (months), BCS (yes/no), sentinel lymph node biopsy (SLNB; yes/no), axillary lymph node dissection (ALND; yes/no), CT (yes/no), and radiotherapy (RT; yes/no). Post-resection histopathological parameters included tumor size (mm), ER, PR, and Ki67 levels (%), and histological grade. Axillary involvement was characterized by the number of sentinel lymph nodes removed, the number of positive sentinel nodes, total lymph nodes removed, and total positive lymph nodes. Follow-up data included recurrence (yes/no) and mortality (yes/no).

This study followed the current classification and treatment strategy for ER-positive/HER2-negative tumors [2,3,7,9] (Figure 1). The PEPI score was calculated as described by Ellis et al. [19] (Table A1).

### 2.3. Statistical Analysis

The statistical analysis was carried out using the IBM SPSS Statistics 29.0 software. Initially, a preliminary analysis of the variables was conducted. Measures of central tendency and dispersion were determined based on the nature of the distribution of each quantitative variable. The mean and standard deviation (SD) were calculated for variables with homogeneous distribution, whereas the median and interquartile range (IQR) were estimated for those with an asymmetric distribution. Categorical variables were provided using frequencies and percentages.

Further analysis was carried out to establish the association between variables. The chi-square test was used for categorical variables, with Fisher’s exact test correction when needed. Association between qualitative and quantitative variables was measured using either Student’s *t*-test or ANOVA, depending on the nature of the categorical variable (dichotomous or ordinal, respectively). The Pearson correlation test was applied for quantitative variables. For those that were considered statistically significant, the magnitude of the association was studied using a single linear regression or a multiple linear regression, determined by the number of independent variables that affect the studied variable (one, two, or more, respectively). Progression-free survival (PFS) and overall survival (OS) were analyzed using Kaplan–Meier analysis and log-rank test. Additionally, Cox regression analysis was also conducted for statistically significant variables.

Statistical associations with a *p*-value < 0.05 were considered significant in the statistical analysis.

## 3. Results

### 3.1. General Overview

This study analyzed data from 175 tumors belonging to a total of 173 patients; in two cases, the additional tumors corresponded to synchronous primaries. Details regarding patient features, tumor features at diagnosis, therapeutic interventions, and tumor features at interim CNB and surgical specimen are summarized in Table 1 and Table 2. For detailed information, please refer to the Supplementary Materials.

### 3.2. Response to NET According to HER2 Expression

When comparing the Ki67 levels at diagnosis with the Ki67 levels at interim CNB, a statistically significant reduction was found (mean: 14.8%, *p* < 0.001), as did when comparing the initial Ki67 levels with the Ki67 levels following resection (mean: 14%, *p* < 0.001). A significant reduction was also observed between the tumor size at diagnosis and the tumor size in the surgical specimen (mean: 11.6 mm, *p* < 0.001). When comparing the decrease in Ki67 levels between initial and interim CNB between the three HER2 groups, no significant differences were found (HER2-zero mean: 16%; HER2-low 1+ mean: 13.5%; HER2-low 2+ mean: 16.1%); nor were they found when contrasting the reduction in Ki67 levels at diagnosis and following resection between these groups (HER2-zero mean: 16.2%; HER2-low 1+ mean: 12.5%; HER2-low 2+ mean: 14.6%), nor when examining tumor size reduction between the three HER2 groups (HER2-zero mean: 14.1 mm; HER2-low 1+ mean: 10.7 mm; HER2-low 2+ mean: 10.9 mm). Lastly, when contrasting the PEPI score value between these three groups, no significant statistical differences were found (HER2-zero mean: 1.3; HER2-low 1+ mean: 1.5; HER2-low 2+ mean: 2.1) (Table 3).

### 3.3. Response to NET According to Other Clinical and Histopathological Tumor Features

Evaluating the association between the variation in Ki67 levels at diagnosis and interim CNB and other clinical and histopathological tumor features, a significant weak direct correlation was found between Ki67 variation and ER levels at diagnosis (r = 0.19, *p* < 0.05). No associations were found between this variation in Ki67 levels and other studied variables (Table 4). A total of 3.8% of the variation between Ki67 levels at diagnosis and interim CNB was explained by the ER levels at diagnosis (R^2^ = 0.038), indicating that higher ER levels were associated with greater variation in Ki67 levels (B = 0.19, *p* < 0.05).

When analyzing the association between the variation in Ki67 levels at diagnosis and following surgical procedure and other clinical and histopathological tumor features, a significant weak direct correlation was observed between Ki67 variation and ER levels at diagnosis (r = 0.24, *p* < 0.05). Significant differences were also found between histological grades at diagnosis (*p* < 0.05), where Ki67 levels had a more pronounced decrease for G3 tumors (mean = 17.8%, 95% CI = 11.7–24.0), followed by G2 tumors (mean = 14.1%, 95% CI = 12.2–16.1) and G1 tumors (mean = 10.2%, 95% CI = 7.1–13.4). No associations were found between this variation in Ki67 levels and other studied variables (Table 4). A total of 9.4% of the variation between Ki67 levels at diagnosis and surgical specimen was explained by the ER levels and histological grade at diagnosis (adjusted-R^2^ = 0.094). The relationship was slightly stronger for the ER levels (B = 0.26, *p* < 0.001) than for histological grade (B = 0.23, *p* < 0.05).

When evaluating the association between tumor size reduction and other clinical and histopathological tumor features, a significant weak indirect correlation was found between the variation in tumor size and Ki67 levels at interim CNB (r = −0.20, *p* < 0.05), as well as a significant weak direct correlation with NET duration (r = 0.22, *p* < 0.05). Significant differences were also observed between histological subtypes regarding reduction in tumor size (*p* < 0.05), where rare carcinomas (mean = 18.8 mm, 95% CI = 2.2–35.5) and lobular carcinomas (mean = 16.8 mm, 95% CI = 8.0–25.6) underwent greater size reduction than ductal carcinomas (mean = 9.6 mm, 95% CI = 8.0–11.2). No associations were found between tumor size variation and other studied variables (Table 4). A total of 3.5% of the variation in tumor size was explained by NET duration (adjusted-R^2^ = 0.035), suggesting that longer NET duration was associated with a greater variation in tumor size (B = 0.21, *p* < 0.05). Assessing the correlation between the PEPI score and other clinical and histopathological tumor features, a significant weak indirect correlation was found between PEPI score and ER and PR levels at diagnosis (r = −0.28, *p* < 0.001; r = −0.17, *p* < 0.05, respectively), and a significant direct weak correlation with Ki67 levels at interim CNB (r = 0.29, *p* < 0.001). Significant differences were also observed between PEPI score and clinical node status (*p* < 0.001), where positive axilla (mean = 3.1) had higher PEPI score values than cN0 patients (mean = 1.3). No associations were found between PEPI score and other studied variables (Table 4). A total of 24.6% of the PEPI score was explained by the clinical node status, ER levels at diagnosis, and Ki67 levels at interim CNB (adjusted-R^2^ = 0.246). The relationship was slightly stronger for the clinical node status (B = 0.39, *p* < 0.001), followed by Ki67 levels at interim CNB (B = 0.20, *p* < 0.05) and ER levels at diagnosis (B = −0.19, *p* < 0.05).

### 3.4. Association Between HER2 Expression (0, 1+ or 2+ in IHC) and Other Clinical and Histopathological Tumor Features

When evaluating the association between HER2 expression and other clinical and histopathological tumor characteristics, including age, clinical node status, histological subtype, ER, PR, and Ki67 levels at diagnosis, tumor size at diagnosis, histological grade at diagnosis, and Ki67 levels at interim CNB, no significant associations were found for any of the studied variables, nor were they observed for these variables when stratifying HER2-zero versus HER2-low tumors.

### 3.5. Progression-Free Survival (PFS) Based on HER2 Expression and Response to NET

**Figure 3:**
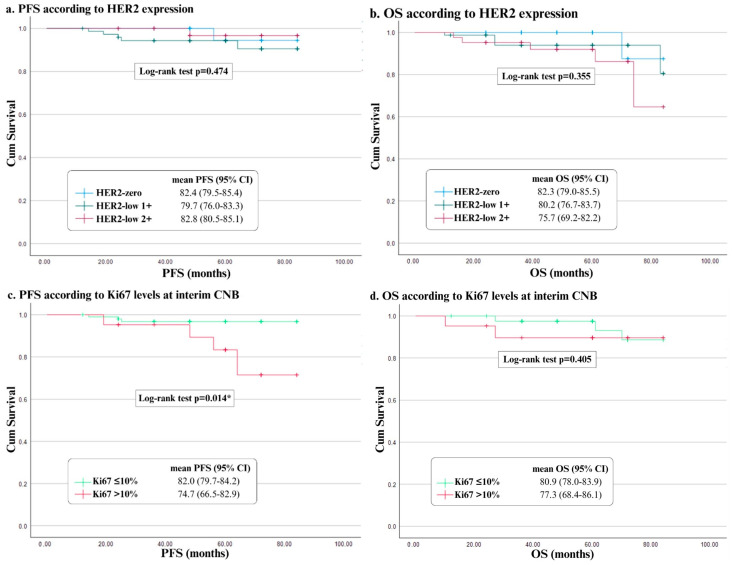
Kaplan-Meyer analysis of PFSand OS according to HER2 expression and Ki67 levels at interim CNB. (**a**) PFS according to HER2 expression. (**b**) OS according to HER2 expression. (**c**) PFS according to Ki67 levels at interim CNB. (**d**) OS according to Ki67 levels at interim CNB. Abbreviations: CNB, core needle biopsy; HER2, human epithelial growth factor receptor; OS, overall survival; PFS, progression-free survival. * *p* values < 0.05 are considered statistically significant. Created with IBM SPSS Statistics 29.0.

Follow-up survival data were available for 154 patients out of the 173 patients included in the study. Recurrence was reported for 7 patients (4.5%), and 11 deaths (7.1%) were documented, of which 10 were non-cancer-related, and 1 was attributed to disease progression. The mean PFS was 81.3 months (95% CI = 79.2–83.3) and the mean overall survival (OS) was 79.4 months (95% CI = 76.7–82.1). No significant differences were found between HER2-zero (mean = 82.4), HER2-low 1+ (mean = 79.7), and HER2-low 2+ (mean = 82.8) in terms of PFS (Figure 3a). No significant differences were found concerning OS when comparing HER2 expression between groups (HER2-zero mean = 82.3, HER2-low 1+ mean = 80.2, HER2-low 2+ mean = 75.7) (Figure 3b). Significant differences were observed (*p* < 0.05) when evaluating recurrence concerning Ki67 percentage at interim CNB, where the group with Ki67 level that dropped to ≤10% at interim CNB (mean = 82.0, 95% CI = 79.7–84.2) had longer PFS than the group whose Ki67 levels at interim CNB were >10% (mean = 74.7, 95% CI = 66.5–82.9) (Figure 3c). However, no significant differences were found when evaluating OS with regard to Ki67 percentage at interim CNB (mean Ki67 ≤ 10% = 80.9, mean Ki67 > 10% = 77.3) (Figure 3d).

When evaluating recurrence concerning response to NET, significant differences were found for the Ki67 level variation between diagnosis and interim CNB (mean difference = 10.9, t = 2.88, *p* < 0.05) and between diagnosis and surgical specimen (mean difference = 9.7, t = 2.12, *p* < 0.05), as well as for PEPI score value (mean difference = −1.4, t = −2.03, *p* < 0.05). However, no significant differences were found for tumor size variation in terms of recurrence. A 15% reduction in the risk of recurrence was observed for greater differences in Ki67 levels between diagnosis and interim CNB (Hazard ratio (HR) = 0.85, 95% CI: 0.76–0.95, *p* < 0.05). When analyzing mortality with regard to response to NET, no significant differences were found for any of the variables. (Table 5).

## 4. Discussion

In recent years, concerns have been raised regarding tumors that express low levels of HER2 and are currently classified as HER2-negative, leading to a significant shift away from the traditional HER2-positive versus HER2-negative stratification. Nevertheless, HER2 testing is highly variable, despite the College of American Pathologists (CAP) guidelines [9]. A few studies have found that HER2 expression fluctuates substantially over time, with HER2-zero tumors converting to HER2-low tumors (15–44%) and vice versa (14–22%) [22,23]. Miglietta et al. [22] noted that HER2 discordance between baseline and relapse samples was increased in ER-positive/HER2-negative tumors when compared to ER-negative/HER2-negative tumors. However, whether this oscillation is due to an actual change in tumor HER2 expression or to acknowledged observer- and methodology-dependent differences remains unknown. Moreover, studies have revealed that HER2-low 1+ and HER2-zero are frequently misdiagnosed, as evidenced by the low agreement between pathologists [24]. This hints at the need for HER2-testing standardization to ensure reliable results and coherent treatment decisions.

In this study, when evaluating clinical and histopathological features of the tumor and comparing HER2-zero, HER2-low 1+, and HER2-low 2+ tumors, no significant differences were observed. There were also no differences in NET response when comparing the three groups. In line with these findings, Wei et al. [24] found no significant differences between HER2-zero and the HER2-low groups in terms of clinical and histopathological characteristics of the tumors. In contrast, some authors have found that HER2-low tumors (including 1+ and 2+) were associated with increased Ki67 values (90.8% versus 62.7%, *p* < 0.001), axillary node positivity (66.5% versus 50.2%, *p* < 0.05), and a lower pathological complete response (pCR) (17.5% versus 23.6%, *p* < 0.05) when compared to HER2-zero tumors [14,25]. Follow-up survival data in this study found no significant differences between the HER2-zero, the HER2-low 1+, and the HER2-low 2+ groups in terms of PFS or OS. In accordance with the mentioned results, similar PFS and OS were observed for the HER2-zero and the HER2-low groups [24]. Gamrani et al. [14] found that HER2-low had shorter PFS than HER2-zero tumors (66% versus 80%, *p* < 0.001). In contrast, Denkert et al. [25] revealed that patients with HER2-low tumors remained disease-free for longer than HER2-zero patients (83.4% vs. 76.1%, *p* < 0.05). Both studies revealed no significant differences between groups in terms of OS [14,25]. A meta-analysis study displayed that HER2-low tumors had longer PFS and OS than the HER2-zero group [26]. These scattered results involving HER2-zero and HER2-low tumors could be explained by HER2 inter-observer and inter-institution testing. More studies with HER2-standardized testing and larger cohorts should be conducted in order to reach reliable conclusions.

The HER2 protein is an important therapeutic target in BC treatment [6,8], and anti-HER2-directed therapy comprises one of the four main pillars of pharmacological BC treatment, alongside endocrine therapy, chemotherapy, and immunotherapy. At present, patients with HER2-enriched tumors (ER-positive/HER2-positive or ER-negative/HER2-positive) are treated with anti-HER2 targeted therapies, such as trastuzumab or pertuzumab, that are often administered in combination with CT [8]. However, tumors classified as HER2-negative in the early-stage setting are treated with either endocrine therapy or CT alone according to the presence or absence of hormone receptors, respectively, but not anti-HER2-directed therapies [2,11]. It is therefore reasonable to suppose that low levels of HER2 expression could be used as a target, even if not the main one. Recently developed antibody–drug conjugates (ADCs) have proved to be a useful tool in the treatment of HER2-low tumors. Trastuzumab-Deruxtecan (T-DXd) is a humanized HER2 monoclonal antibody bound to a topoisomerase I inhibitor, an ensemble that allows for the chemotherapeutic agent to reach specific HER2-enriched cells. It also has an off-target effect that enhances efficacy by attacking neighboring tumor cells. The DESTINY-Breast04 trial revealed longer PFS for the T-DXd ER-positive/HER2-low group (10.1 months) than for the physician’s choice of therapy (5.4 months), as well as greater OS (23.9 months vs. 17.5 months, respectively), in the metastatic setting [21]. There are other ongoing clinical trials researching different treatment options for HER2-low BC, such as other monoclonal antibodies, ADCs, tumor vaccines, bi/tri-specific antibodies, and tyrosine kinase inhibitors [6].

On a related note, NET is a very attractive treatment alternative for postmenopausal women with low-grade luminal HER2-negative tumors [2]. Unfortunately, no definitive endpoints have been approved for NET response [12]. The most commonly used biomarker is Ki67 levels at interim biopsy (2–4 weeks after initiating NET), where a decrease in Ki67 levels below 10% is regarded as an appropriate response to endocrine therapy [17]. The PEPI score and tumor size are also employed to establish prognosis for luminal tumors after NET [12,19,20]. In accordance with this data, the differences in Ki67 expression between diagnosis, interim CNB, and surgical specimen, as well as tumor size variation, were all found to be statistically significant in this study. These findings suggest that these parameters serve as valid endpoints for assessing NET response. In search for other biomarkers that could be useful to determine NET response, residual cancer burden, and pCR are frequently used endpoints for NCT, although luminal tumors have shown low pCR rates for both NCT (when compared to ER-negative tumors) and NET [12,20]. Further studies are needed to determine additional specific biomarkers to aid in predicting NET response and prognosis.

In this study, significant differences were found across the four studied variables for NET response and different clinical and histopathological tumor features. Higher ER levels at diagnosis were linked to a greater difference in Ki67 levels between diagnosis, interim CNB, and surgical specimen. In line with these results, lower ER levels were associated with a higher PEPI score. A higher histological grade at diagnosis was linked to increased variation in Ki67 levels between diagnosis and surgical specimen. High Ki67 levels at interim CNB were linked to a higher PEPI score. A clinically positive axilla was also linked to a higher PEPI score, although this could be attributed both to the low rates of axillary downstaging typically associated with neoadjuvant therapy and to the substantial weight that nodal involvement carries in the PEPI scoring system. These results are all in accordance with previously known features that contribute to NET response [16,19,27]. Longer NET duration was also associated with greater variation in tumor size, suggesting that prolonging this therapy may achieve better results [16]. When evaluating Ki67 levels at interim CNB, the group with Ki67 levels that dropped to ≤10% had a significantly longer PFS than the group whose levels did not decrease to said mark. PFS was also associated with a wider variation in Ki67 levels between diagnosis, interim CNB, and surgical specimen, and a lower PEPI score. These findings remain in line with conclusions from other studies [17,19]. No significant differences were found when evaluating overall survival, which could be explained by the short follow-up duration of the study. To confirm this, another study should be conducted with a longer tracking period.

To our knowledge, this is the first study to analyze NET response according to HER2 expression. Given that low levels of HER2 expression do not seem to meaningfully affect the response to NET in ER-positive/HER2-negative BC when compared to HER2-zero tumors, our findings do not support a change in the current clinical practice for patients with HER2-low tumors (be it 1+ or 2+) in the NET setting. These results raise the question of whether higher levels of HER2 expression may similarly have a limited impact on NET response in ER-positive/HER2-positive tumors. However, the relationship between HER2 expression and NET response remains unclear. Many authors have discussed an existing bidirectional crosstalk between the ER and HER2 intracellular signaling pathways. HER2 overexpression leads to inhibition of ER expression and cell proliferation. On the other hand, ER activation results in an upregulation of HER2 expression, both through genomic and non-genomic pathways [28,29]. Congruent with these findings, HER2-low tumors are consistently more frequent in luminal tumors than ER-negative tumors [22,23]. This crosstalk could further explain HER2 variation over time. Moreover, resistance to endocrine therapy has also been considered as a consequence of the overlap between these signaling pathways for HER2-positive and HER2-low tumors [8,10,28]. Some authors have suggested that targeting HER2 may induce a compensatory increase in ER signaling, potentially promoting tumor proliferation and treatment resistance [29]. Building upon this idea, Park et al. [30] combined NET and neoadjuvant anti-HER2 targeted therapy in luminal HER2-positive breast cancer, with controversial results. Further studies with larger cohorts should be conducted to establish whether this could be a form of treatment for patients with luminal BC that overexpress HER2 protein to any extent, including HER2-low and HER2-positive tumors.

This real-world, single-centered study ensured sample homogeneity, enhancing the reliability of comparisons and minimizing confounding. However, limitations include its retrospective design, small sample size, and short follow-up, which warrant cautious interpretation—particularly of PFS and OS outcomes. Additionally, complete staging data (ypT and ypN) were not available for all 173 patients, as some final surgical pathology reports did not specify tumor size or the exact number of affected lymph nodes. This missing data may slightly limit the granularity of our outcome analysis.

Our findings suggest that low HER2 expression does not significantly influence response to neoadjuvant endocrine therapy (NET) in luminal tumors compared to HER2-zero cases, nor does it impact other clinical or histopathological features. Instead, NET response may be influenced by factors such as ER levels, histological subtype and grade, and early Ki67 changes. A decrease in Ki67 after short-term NET may be associated with longer PFS. Larger prospective studies with extended follow-up are needed to validate these findings and better understand predictors of response.

## Figures and Tables

**Table 1:** Patient and tumor features at diagnosis.

		HER2 Status			Overall
		HER2-Zero	HER2-Low 1+	HER2-Low 2+	
		Median (IQR)	Median (IQR)	Median (IQR)	Median (IQR)
Age (years)		71 (63.0–76.0)	68.0 (63.0–78.2)	72.0 (63.0–81.0)	70.0 (63.0–79.0)
Tumor size (mm)		25.0 (17.0–32.2)	25.0 (18.0–30.0)	25.0 (17.0–35.0)	25.0 (17.0–33.0)
ER expression (%)		100.0 (100.0–100.0)	100.0 (100.0–100.0)	100.0 (100.0–100.0)	100.0 (100.0–100.0)
PR expression (%)		80.0 (40.0–100.0)	70.0 (15.0–100.0)	70.0 (15.0–90.0)	70.0 (20.0–100.0)
Ki67 levels (%)		20.0 (10.0–25.0)	17.0 (12.0–25.5)	22.0 (15.0–30.0)	20.0 (12.0–30.0)
		***n* (%)**	***n* (%)**	***n* (%)**	***n* (%)**
Clinical primary tumor (cT)	≤20 mm (cT1)	14 (33.3%)	34 (40.5%)	17 (34.7%)	65 (37.2%)
	>20 and ≤50 mm (cT2)	25 (59.5%)	43 (51.2%)	29 (59.2%)	97 (55.4%)
	>50 mm (cT3)	3 (7.2%)	4 (4.7%)	3 (6.1%)	10 (5.7%)
	DNA	-	3 (3.6%)	-	3 (1.7%)
Clinical node status (cN)	Negative (cN0)	35 (83.3%)	69 (82.1%)	36 (73.5%)	134 (80.0%)
	Positive (cN1–N3)	5 (11.9%)	12 (14.3%)	12 (24.5%)	29 (16.6%)
	DNA	2 (4.8%)	3 (3.6%)	1 (2.0%)	6 (3.4%)
Histological subtype	Ductal	31 (73.8%)	61 (72.6%)	39 (79.6%)	131 (74.9%)
	Lobular	5 (11.9%)	18 (21.4%)	7 (14.3%)	30 (17.1%)
	Others	6 (14.3%)	5 (6.0%)	3 (6.1%)	14 (8.0%)
Histological grade	1	10 (23.8%)	13 (15.5%)	10 (20.4%)	33 (18.8%)
	2	30 (71.4%)	53 (63.1%)	34 (69.4%)	117 (66.9%)
	3	2 (4.8%)	18 (21.4%)	5 (10.2%)	25 (14.3%)

Abbreviations: DNA—data non-available; ER—estrogen receptor; HER2—human epidermal growth factor receptor 2; IQR—interquartile range; PR—progesterone receptor.

**Table 2:** Therapeutic interventions and tumor features at interim CNB and surgical specimen.

		HER2 Status			
		HER2-Zero	HER2-Low 1+	HER2-Low 2+	
		Median (IQR)	Median (IQR)	Median (IQR)	Median (IQR)
NET duration (months)		5.0 (3.0–7.0)	6.0 (3.0–7.0)	6.0 (3.7–7.0)	6.0 (3.0–7.0)
Interim CNB	ER expression (%)	100.0 (100.0–100.0)	100.0 (100.0–100.0)	100.0 (95.0–100.0)	100.0 (100.0–100.0)
	PR expression (%)	27.0 (4.0–72.5)	0.0 (0.0–27.5)	5.0 (0.0–40.0)	5.0 (0.0–40.0)
	Ki67 levels (%)	3.0 (1.0–5.0)	5.0 (1.0–10.0)	5.0 (2.0–8.5)	5.0 (2.0–8.5)
SS	ER expression (%)	100.0 (100.0–100.0)	100.0 (100.0–100.0)	100.0 (100.0–100.0)	100.0 (100.0–100.0)
	PR expression (%)	5.0 (0.0–25.0)	0.0 (0.0–5.0)	2.0 (0.0–5.0)	0.0 (0.0–15.0)
	Ki67 levels (%)	1.0 (1.0–3.0)	2.0 (1.0–10.0)	3.0 (1.0–10.0)	2.0 (1.0–7.0)
SS tumor size (mm)		12.0 (8.0–19.2)	15.0 (12.0–20.0)	15.0 (9.5–20.0)	15.0 (10.0–20.0)
PEPI score		0.5 (0.0–3.0)	1.0 (0.0–3.0)	2.0 (0.0–4.0)	1.0 (0.0–3.0)
Axillary surgery	Sentinel LN extracted (*n*)	2.0 (1.0–2.0)	1.0 (1.0–2.0)	2.0 (1.0–3.0)	2.0 (1.0–3.0)
	Positive sentinel LN (*n*)	0.0 (0.0–1.0)	0.0 (0.0–0.0)	0.0 (0.0–1.0)	0.0 (0.0–1.0)
	Total LN extracted (*n*)	2.0 (2.0–3.0)	6.0 (2.0–9.5)	3.5 (2.0–9.0)	3.0 (2.0–7.0)
	Total positive LN (*n*)	0.5 (0.0–1.0)	1.0 (0.0–3.0)	1.5 (0.0–3.2)	1.0 (0.0–1.0)
		***n* (%)**	***n* (%)**	***n* (%)**	***n* (%)**
Histological grade at interim CNB	1	8 (19.0%)	17 (20.2%)	11 (22.5%)	36 (20.6%)
	2	23 (54.8%)	45 (53.6%)	25 (51.0%)	93 (53.1%)
	3	0 (0.0%)	3 (3.6%)	1 (2.0%)	4 (2.3%)
	DNA	11 (26.2%)	19 (22.6%)	12 (24.5%)	42 (24.0%)
Histological grade in SS	1	19 (45.2%)	34 (40.5%)	17 (34.7%)	70 (40.0%)
	2	19 (45.2%)	43 (51.2%)	25 (51.0%)	87 (49.7%)
	3	1 (2.4%)	5 (6.0%)	4 (8.2%)	10 (5.7%)
	DNA	3 (7.2%)	2 (2.3%)	3 (6.1%)	8 (4.6%)
Pathological primary tumor (ypT)	≤20 mm (ypT1)	31 (73.8%)	62 (73.8%)	37 (75.5%)	130 (74.3%)
	>20 and ≤50 mm (ypT2)	8 (19.0%)	18 (21.4%)	9 (18.4%)	35 (20.0%)
	>50 mm (ypT3)	1 (2.4%)	0 (0.0%)	1 (2.0%)	2 (1.1%)
	DNA	2 (4.8%)	4 (4.8%)	2 (4.1%)	8 (4.6%)
Pathological node status (ypN)	0 lymph nodes (ypN0)	27 (60.0%)	54 (63.5%)	21 (46.6%)	102 (58.3%)
	1–3 lymph nodes (ypN1)	11 (24.4%)	15 (17.6%)	17 (37.8%)	43 (24.6%)
	4–9 lymph nodes (ypN2)	0 (0.0%)	5 (5.9%)	3 (6.7%)	8 (4.6%)
	≥10 lymph nodes (ypN3)	0 (0.0%)	1 (1.2%)	1 (2.2%)	2 (1.1%)
	DNA	7 (15.6%)	10 (11.8%)	3 (6.7%)	20 (11.4%)
Breast intervention	BCS	38 (21.7%)	69 (39.4%)	41 (23.4%)	148 (84.6%)
	Mastectomy	4 (2.3%)	15 (8.6%)	8 (4.6%)	27 (15.4%)
Axillary intervention	SLNB	38 (21.7%)	70 (40.0%)	39 (22.3%)	147 (84.0%)
	ALND	2 (1.1%)	15 (8.6%)	6 (3.4%)	23 (13.1%)
Adjuvant treatment	RT	36 (20.6%)	68 (38.9%)	41 (23.4%)	145 (82.9%)
	CT	6 (3.4%)	18 (10.3%)	9 (5.1%)	33 (18.9%)

Abbreviations: ALND—axillary lymph node dissection; BCS—breast conserving surgery; CNB—core needle biopsy; CT—chemotherapy; DNA—data non-available; ER—estrogen receptor; IQR—interquartile range; LN—lymph nodes; NET—neoadjuvant endocrine therapy; PEPI—Preoperative Endocrine Prognostic Index; PFS—progression-free survival; PR—progesterone receptor; RT—radiotherapy; SLNB—selective sentinel lymph node biopsy; SS—surgical specimen.

**Table 3:** Response to NET according to HER2 expression.

	Mean	*p*	Mean			*p*
	Total		HER2-Zero	HER2-Low 1+	HER2-Low 2+	
Difference in Ki67 levels (diagnosis—interim CNB) (%)	14.8	0.0001 *	16	13.5	16.1	0.335
Difference in Ki67 levels (diagnosis—SS) (%)	14.0	0.0001 *	16.2	12.5	14.6	0.196
Variation in tumor size (diagnosis—SS) (mm)	11.6	0.0001 *	14.1	10.7	10.9	0.477
PEPI score			1.3	1.5	2.1	0.059

* *p* values < 0.05 are considered statistically significant. Abbreviations: CNB—core needle biopsy; HER2—human epidermal growth factor receptor; NET—neoadjuvant endocrine therapy; PEPI—Preoperative Endocrine Prognostic Index; SS—surgical specimen.

**Table 4:** Response to NET according to other tumor features.

	Difference in Ki67 Levels (Diagnosis—Interim CNB)		Difference in Ki67 Levels (Diagnosis—SS)		Variation in Tumor Size (Diagnosis—SS)		PEPI Score	
	r/F/t	*p*	r/F/t	*p*	r/F/t	*p*	r/F/t	*p*
Age (years)	−0.06	0.506	0.08	0.306	0.09	0.274	−0.07	0.373
Clinical node status (cN)	0.45	0.656	1.36	0.177	−1.94	0.055	−5.41	0.0001 *
Histological subtype	0.34	0.712	2.19	0.116	4.71	0.01 *	0.77	0.465
ER expression (%) at diagnosis	0.19	0.025 *	0.24	0.003 *	0.02	0.781	−0.28	0.0001 *
PR expression (%) at diagnosis	0.08	0.351	0.15	0.062	0.12	0.116	−0.17	0.029 *
Histological grade at diagnosis	2.52	0.085	3.32	0.039 *	0.29	0.749	0.15	0.859
Ki67 levels (%) at interim CNB					−0.20	0.025 *	0.29	0.001 *
NET duration (months)	0.14	0.104	−0.02	0.829	0.22	0.004 *	−0.6	0.402

* *p* values < 0.05 are considered statistically significant. Abbreviations: CNB—core needle biopsy; ER—estrogen receptor; NET—neoadjuvant endocrine therapy; PEPI—Preoperative Endocrine Prognostic Index; PR—progesterone receptor; SS—surgical specimen.

**Table 5:** Outcomes according to response to NET.

	Recurrence				Mortality			
	*n*	Mean Difference (CI †)	t	*p*	*n*	Mean Difference (CI †)	t	*p*
Difference in Ki67 levels (diagnosis—interim CNB) (%)	7	10.9 (3.4–18.5)	2.88	0.005 *	6	6.2 (−2.1–14.5)	1.47	0.144
Difference in Ki67 levels (diagnosis—SS) (%)	6	9.7 (0.7–18.8)	2.12	0.035 *	10	4.5 (−2.7–11.7)	1.22	0.223
Variation in tumor size (diagnosis—SS) (mm)	7	8.6 (−3.0–20.2)	1.47	0.143	11	8.2 (−1.1–17.6)	1–74	0.084
PEPI score	7	−1.4 (−2.8–(−0.04))	−2.03	0.044 *	11	−0.5 (−1.7–0.6)	−0.97	0.332

* *p* values < 0.05 are considered statistically significant. † CI at 95%. Abbreviations: CNB—core needle biopsy; PEPI—Preoperative Endocrine Prognostic Index; SS—surgical specimen.

## Data Availability

The data presented in this study are available on request from the corresponding author.

## Supplementary Materials

The following supporting information can be downloaded at: https://www.mdpi.com/article/10.3390/curroncol33020099/s1, General Overview of patient and tumor features at diagnosis (Table S1); General overview of therapeutic interventions and tumor features at interim CNB and surgical specimen (Table S2).

## Abbreviations

ADC	Antibody–drug conjugate
ALND	Axillary lymph node dissection
BC	Breast cancer
BCS	Breast-conserving surgery
CAP	College of American Pathologists
CNB	Core needle biopsy
CT	Chemotherapy
ER	Estrogen receptors
ET	Endocrine therapy
HER2	Human epidermal growth factor receptor 2
HULP	Hospital Universitario La Paz
IDC	Invasive ductal carcinoma
IHC	Immunohistochemistry
ILC	Invasive lobular carcinoma
IQR	Interquartile range
ISH	In situ hybridization
NAT	Neoadjuvant therapy
NCT	Neoadjuvant chemotherapy
NET	Neoadjuvant endocrine therapy
OS	Overall survival
pCR	Pathological complete response
PEPI	Preoperative Endocrine Prognostic Index
PFS	Progression-free survival
PR	Progesterone receptors
RT	Radiotherapy
SD	Standard deviation
SLNB	Selective sentinel lymph node biopsy
T-DXd	Trastuzumab-deruxtecan

## Appendix A

**Table A1:** PEPI score.

		Points
Tumor size	pT1–pT2 (<50 mm)	0
	pT3–pT4 (>50 mm)	3
Node status	Negative (pN0)	0
	Positive (pN1–pN3)	3
Ki67 level at SS	0–2.7%	0
	2.7–19.7%	1
	19.7–53.1%	2
	>53.1%	3
ER	0–2	3
	3–8	0

The PEPI score evaluates surgical specimen data, assigning a specific score according to tumor size, node status, ER status, and Ki67 levels categories. Abbreviations: ER—estrogen receptors; PEPI—Preoperative Endocrine Prognostic Index; SS—surgical specimen. Adapted from: Ellis et al. [19].
