# Supplementary material for: Role of HER2 in Response to Neoadjuvant Endocrine Therapy in Luminal Breast Cancer
Source: Curr Oncol. 2026 Feb 4;33(2):99. doi: 10.3390/curroncol33020099 (PMC12939190; doi:10.3390/curroncol33020099)
Supplement: Supplementary file 1 [file curroncol-33-00099-s001.zip › curroncol-4047229-supplementary.pdf]

Supplementary File

General Overview of patient and tumor features at diagnosis (Table S1)

Participants had a median age of 70 years (IQR 63-79), ranging from a minimum of 50 years to a maximum of 90. Regarding tumor radiological features at diagnosis, the median tumor size was 25 mm (IQR 17-33) and 16.6% (n=29) of the patients had a lymph-node positive axilla (cN1-N3). The most frequent subtype, encompassing 74.9% (n=131) of all tumors, was the IDC; followed by the ILC (17.1%) and other rare subtypes (8%). Of the total, 24.0% (n=42) of the tumors were HER2-zero (IHC score 0), 48.0% (84) were HER2-low 1+, and 28.0% (n=49) were HER2-low 2+. Moderately differentiated (G2) carcinomas comprised 66.9% (n=117) of all tumors, with low grade (G1) and high grade (G3) carcinomas accounting for 18.9% and 14.3% respectively. The median initial Ki67 value was 20% (IQR 12-30). For ER and PR levels, median and IQR values were 100% (100-100) and 70% (20-100), respectively.

General overview of therapeutic interventions and tumor features at interim CNB and surgical specimen (Table S2)

Patients were treated with NET for a median period of 6 months (IQR 3-7). For those patients with an interim CNB (n=133), the median Ki67 value was 5% (IQR 2-8.5), and G2 carcinomas encompassed 69.9% (n=93) of tumors followed by G1 tumors (27.1%). BCS was performed in 84.6% (n=148) of patients, whereas mastectomy was conducted in the remaining 15.4% (n=27), primarily due to factors such as multifocal disease, poor tumor-to-breast size ratio, or patient preference. In relation to axillary surgery, SLNB was the primary intervention performed, as opposed to ALND, with rates of 84.0% (n=147) and 13.1% (n=23) respectively. The average tumor size following surgery was 15mm (IQR 10-20). G2 carcinomas accounted for 49.7% (n=87) of all tumors, followed by G1 and G3 carcinomas (40% and 5.7%, respectively). The final median Ki67 value in the surgical specimen was 2% (IQR 1-7). For ER and PR levels at surgical specimen, median and IQR values were 100% (100-100) and 0% (0-15), respectively. In relation to pathological node status, 58.3% (n=102) of patients were ypN0, and 24.6% had positive lymph nodes (ypN1-3). Median PEPI-score value was 1 (IQR 0-3). Most patients received some form of adjuvant treatment, in addition to hormone therapy, including RT in 82.9% (n=145) of the subjects, and QT in 18.9% (n=33).
